# Supplementary material for: Exploration of the decontamination of common nonmetallic materials by Ce(IV)/HNO3
Source: PLoS One. 2025 Sep 8;20(9):e0322683. doi: 10.1371/journal.pone.0322683 (PMC12416749; doi:10.1371/journal.pone.0322683)
Supplement: S1 — (DOCX) [file pone.0322683.s001.docx]

**2. pH Influence(inferred from HNO₃ concentration**

Despite fixed HNO₃ concentrations, pH effects were inferred:

Quartz: DF peaked at pH ~1.5 (1.5 mol/L HNO₃), consistent with Ce(IV) stability window [41]

Ceramic: Optimal pH ~1.0 (2.0 mol/L HNO₃) due to:

Al₂O₃ dissolution at low pH [42]

Suppressed Ce(III) precipitation (pH < 2.5) [43]
