# Supplementary material for: Exploration of the decontamination of common nonmetallic materials by Ce(IV)/HNO3
Source: PLoS One. 2025 Sep 8;20(9):e0322683. doi: 10.1371/journal.pone.0322683 (PMC12416749; doi:10.1371/journal.pone.0322683)
Supplement: S2 — (DOCX) [file pone.0322683.s002.docx]

**3. Comparative Efficiency**

The kinetic advantage of ceramics diminishes at pH > 2 due to:

Ce(IV) hydrolysis [44-47]

Iodine re-adsorption on proton-depleted surfaces

**Table S1. Time-dependent decontamination factors (DF) for quartz glass**

| **Time**  **(min)** | **20°C**  **(0.01M Ce(IV), 1.0M HNO₃** | **60°C**  **(0.02M Ce(IV), 1.5M HNO₃** | **80°C**  **(0.1M Ce(IV), 2.0M HNO₃)** |
| --- | --- | --- | --- |
| 5 | 1.05 ± 0.04 | 1.32 ± 0.05 | 1.28 ± 0.05 |
| 15 | 1.62 ± 0.06 | 2.85 ± 0.11 | 3.12 ± 0.12 |
| 30 | 2.10 ± 0.08 | 4.73 ± 0.18 | 5.94 ± 0.23 |
| 60 | 2.52 ± 0.10 | 6.83 ± 0.26 | 8.14 ± 0.31 |
| 120 | 2.55 ± 0.10 | 6.88 ± 0.26 | 8.10 ± 0.31 |

**Table S2. Time-dependent DF for ceramic**

| **Time**  **(min)** | **20°C**  **(0.01M Ce(IV), 1.0M HNO₃** | **80°C**  **(0.02M Ce(IV), 2.0M HNO₃** |
| --- | --- | --- |
| 5 | 1.12 ± 0.04 | 2.57 ± 0.10 |
| 15 | 2.03 ± 0.08 | 8.92 ± 0.34 |
| 30 | 3.57 ± 0.14 | 15.33 ± 0.58 |
| 60 | 3.73 ± 0.14 | 19.52 ± 0.74 |
| 120 | 3.70 ± 0.14 | 18.91 ± 0.72 |
