# Supplementary material for: Exploration of the decontamination of common nonmetallic materials by Ce(IV)/HNO3
Source: PLoS One. 2025 Sep 8;20(9):e0322683. doi: 10.1371/journal.pone.0322683 (PMC12416749; doi:10.1371/journal.pone.0322683)
Supplement: Attachment — Submitted filename: Response_to_Reviewers_PONE-D-25-15932_PAN_20250516.pdf.docx [file pone.0322683.s003.docx]

> *Dear Reviewers,*

> We sincerely appreciate your constructive comments. Revisions include:

> 1. Clarified material selection rationale and L16(4⁴) design (Section 1–2).

> 2. Added adsorption mechanisms and validation methods (Sections 3.1, 2.1).

> 3. Discussed real-world scalability (Conclusion).

> 4. Proposed RSM/ANOVA for future work (Section 2.4).

>**Changes made in the revised manuscript are highlighted in red text for easy identification.**

**1. Reviewer #1 - Question 1**

**Question:** Why were quartz glass and ceramics chosen? Were other nonmetallic materials considered? Why was the L16(4⁴) orthogonal array selected?

**Answe:**

Quartz glass (SiO₂ ≥ 99.9%) and ceramics (85% Al₂O₃, 10% Mg-silicate) were selected due to their widespread use in nuclear medicine facilities and their distinct chemical compositions, which allowed for comparative analysis of decontamination mechanisms. Other nonmetallic materials (e.g., polymers) were not tested but could be explored in future studies. The L16(4⁴) orthogonal array was chosen for its balanced design, enabling efficient investigation of four factors (temperature, Ce(IV)/HNO₃ concentrations, time) at four levels with minimal experimental runs, while maintaining statistical robustness.

**2. Reviewer #1 - Question 2**

**Question:** Is the 4-hour peak adsorption consistent with prior studies? What is the interaction mechanism?

**Answer:**

The 4-hour peak adsorption aligns with studies on iodine-131’s affinity for silica/alumina surfaces (e.g., Huang et al., 2012). The mechanism involves physisorption (van der Waals forces) and chemisorption (ion exchange with surface hydroxyl groups), particularly pronounced in ceramics due to Al₂O₃’s porous structure.

**3. Reviewer #1 - Question 3**

**Question:** Why does DF plateau at 10 mm liquid height? Why do quartz and ceramics favor different HNO₃ concentrations?

**Answer:**

The plateau suggests diffusion equilibrium is reached at 10 mm, minimizing further efficiency gains. Quartz glass favors lower HNO₃ (1.5 mol/L) due to its inert SiO₂ surface, whereas ceramics require higher HNO₃ (2.0 mol/L) to dissolve Al₂O₃-related surface complexes.

**4. Reviewer #1 - Question 5**

**Question:** Were quantitative roughness measurements (AFM) performed? How was iodine-131 validated beyond EDS?

**Answer:**

AFM was not used, but SEM qualitatively confirmed surface cleanliness. Iodine-131 validation relied on β-counting (LB-4 detector), with cross-checked background subtraction (Equation 1), as EDS lacks sensitivity for trace iodine.

**5. Reviewer #2 - Question 1**

**Question:** How do the 4-hour adsorption and optimal conditions translate to real-world applications?

**Answer:**

The 4-hour adsorption simulates accidental contamination scenarios in nuclear facilities. Optimal conditions (e.g., 80°C for ceramics) are scalable to industrial decontamination, though larger objects may require adjusted parameters (e.g., longer time).

**6. Reviewer #2 - Question 3**

**Question:** Clarify the mechanism of organic substance removal.

**Answer:** Ce(IV) oxidizes organic residues via radical reactions (•OH generation), while HNO₃ dissolves inorganic deposits. This dual action is evident in SEM images (Figures 4–5).

**7. Reviewer #2 - Question 5**

**Question:** Why not use RSM/ANOVA for optimization?

**Answer:** Orthogonal tests with extreme difference analysis were chosen for simplicity and direct factor ranking. However, RSM/ANOVA could be valuable for future nonlinear optimization.
