# Supplementary material for: Exploration of the decontamination of common nonmetallic materials by Ce(IV)/HNO3
Source: PLoS One. 2025 Sep 8;20(9):e0322683. doi: 10.1371/journal.pone.0322683 (PMC12416749; doi:10.1371/journal.pone.0322683)
Supplement: Attachment — Submitted filename: Review comments.docx [file pone.0322683.s004.docx]

**Dear Prof. [Editor/Editorial Office] of the Journal of PLOS ONE,**

**Manuscript ID: PONE-D-25-15932**

**This manuscript presents a well-structured and scientifically rigorous exploration of Ce(IV)/HNO₃ decontamination for iodine-131-contaminated nonmetallic materials, offering valuable insights into optimal conditions for quartz glass and ceramics. The orthogonal experimental design is robust, and the combination of SEM/EDS analysis with decontamination factor (DF) metrics provides compelling evidence for the method’s efficacy. The findings are highly relevant to nuclear medicine and radiological safety, addressing a practical need for efficient decontamination strategies. While minor clarifications (e.g., statistical validation, alternative detection methods) could further strengthen the work, the study is methodologically sound, clearly presented, and merits publication after addressing the next comments. Thank you for the opportunity to review this impactful research—it contributes meaningfully to the field and demonstrates both innovation and applicability.**

1- The study aims to explore the decontamination of nonmetallic materials using Ce(IV)/HNO₃. Could the authors elaborate on why these specific materials (quartz glass and ceramics) were chosen, given their widespread use in nuclear medicine? Are there other nonmetallic materials that could benefit from this decontamination method? The orthogonal experimental design is a key component of this study. Could the authors provide more details on why an L16(4⁴) orthogonal array was selected? Were other experimental designs considered, and if so, why were they rejected?

2- Both materials reached peak iodine-131 adsorption at 4 hours. Is this consistent with previous studies, or does it suggest a unique interaction between iodine-131 and these substrates? and what is the mechanism of the interaction?

3- The decontamination factor (DF) plateaued at 10 mm liquid height. Does this imply that diffusion limitations are negligible beyond this point, or are there other factors at play? The best DF for quartz glass was achieved at 60°C, 0.02 mol/L Ce(IV), and 1.5 mol/L HNO₃. Why does quartz glass favor lower HNO₃ concentrations compared to ceramics? Ceramics showed the highest DF at 80°C, 0.02 mol/L Ce(IV), and 2.0 mol/L HNO₃. Does the higher Al₂O₃ content in ceramics influence this preference for stronger acidity?

5- The SEM images show cleaner surfaces post-decontamination. Were any quantitative roughness measurements (e.g., AFM) performed to corroborate these observations? Since EDS could not detect iodine-131, how was the decontamination efficiency validated beyond DF calculations? Were radioactivity measurements cross-checked with other methods?

6- Ce(IV) is known to reduce to Ce(III) in acidic media. Did the authors monitor Ce(IV) concentration over time to ensure consistent oxidizing power during decontamination? Beyond providing acidity, does HNO₃ participate directly in the redox reaction with iodine-131, or does it solely maintain Ce(IV) stability?

7- For ceramics, a shorter decontamination time (1 h) at higher temperature (80°C) was optimal. Could a longer time at lower temperature achieve similar results, or is kinetics the limiting factor? Quartz glass (SiO₂) and ceramics (Al₂O₃/Mg-silicate) have different compositions. How do these differences influence iodine adsorption and subsequent decontamination mechanisms?

8- SEM suggests that organic contaminants were also removed. Were these impurities introduced during contamination, or are they inherent to the materials? Were the orthogonal experiments repeated to assess reproducibility? If not, how confident are the authors in the robustness of the optimal conditions?

9- The DF values vary significantly across test conditions (e.g., 1.51 to 8.14 for quartz). Are these variations due to experimental error, or do they reflect true material-response differences? Could other oxidants (e.g., permanganate, ozone) achieve similar or better decontamination? Why was Ce(IV) chosen over these alternatives?

10- The study fixes HNO₃ concentration but does not discuss pH. Could pH adjustments further optimize decontamination, or is Ce(IV) activity pH-independent in this range? The study focuses on equilibrium conditions (fixed time points). Were any kinetic studies performed to model the rate of iodine removal?

11- The manuscript includes SEM images but does not quantify the changes. Could the authors provide a qualitative description of the differences between pre- and post-decontamination surfaces? The experiments use small samples (φ45 mm). How might decontamination efficiency change for larger or irregularly shaped objects?

12- The introduction section needs to be strengthened by providing a clearer discussion of the study's novelty and positioning it within the context of existing research. How does this work offer a significant improvement or unique contribution compared to previous studies? The authors should emphasize these aspects and support their claims with relevant references to enhance the manuscript’s credibility and depth [<https://doi.org/10.1021/acsapm.4c03794> & <https://doi.org/10.1007/s10967-024-09667-4> & https://doi.org/10.3390/nano12132305].
