# Supplementary material for: Exploration of the decontamination of common nonmetallic materials by Ce(IV)/HNO3
Source: PLoS One. 2025 Sep 8;20(9):e0322683. doi: 10.1371/journal.pone.0322683 (PMC12416749; doi:10.1371/journal.pone.0322683)
Supplement: Attachment — Submitted filename: Response to Reviewer.docx [file pone.0322683.s005.docx]

**Response to Reviewer Comments**

**Manuscript ID: PONE-D-25-15932**

**Title:** Exploration of the decontamination of common nonmetallic materials by Ce(IV)/HNO₃

**Response to Reviewer #1**

We sincerely appreciate the reviewer’s insightful comments and have addressed each point below. Changes in the revised manuscript are highlighted in track changes.

**Comment 1: Material Selection and Orthogonal Design**

**Reviewer’s Question:**

- Why were quartz glass and ceramics chosen? Are other nonmetallic materials applicable?

- Why was the L16(4⁴) orthogonal array selected? Were other designs considered?

**Response:**

**1. Material Selection Rationale:**

Quartz glass (SiO₂) and ceramics (Al₂O₃/Mg-silicate) were selected due to their:

- **Prevalence in Nuclear Medicine**: Quartz is used in radiation shielding windows; ceramics are common in reactor linings (Refs. 9–13).

-**Chemical Stability:** Low reactivity with iodine-131 allows focused study of decontamination mechanisms.

- **Structural Contrast:** Amorphous SiO₂ vs. porous Al₂O₃ enables comparative analysis.

Future studies will extend this method to polymers/concrete (added to Introduction, Lines 45–48).

2. **Orthogonal Design Justification:**

The L16(4⁴) array was chosen for:

**-Efficiency**: Evaluates 4 factors (temperature, Ce(IV), HNO₃, time) at 4 levels with only 16 runs.

**- Statistical Validity:** Balanced and orthogonal (Galois field theory, Ref. 39).

Alternative designs (e.g., full factorial) were rejected due to excessive resource requirements (added to Section 2.4).

**Comment 2: Adsorption Time and Mechanism**

**Reviewer’s Question:**

Is the 4-hour peak adsorption consistent with prior studies? What is the interaction mechanism?

**Response:**

**Consistency:** The 4-hour peak aligns with studies on iodine-131 binding to silica/alumina surfaces (Refs. 9, 13).

**Mechanism:**

- **Quartz Glass:** Physisorption via Si-OH groups.

- **Ceramics:** Ion exchange at Al-OH/Mg-silicate sites (added to Section 3.1, Table 10).

**Comment 3: DF Plateau and Material-Specific HNO₃ Dependence**

**Reviewer’s Question:**

Does the DF plateau at 10 mm imply diffusion limits? Why does quartz favor lower HNO₃ than ceramics?

**Response:**

**1. DF Plateau:**

Beyond 10 mm, diffusion limitations become negligible (Fick’s law, Section 3.2).

**2. HNO₃ Dependence:**

**Quartz:** Optimal at 1.5 mol/L HNO₃ (pH ~1.5) to preserve Ce(IV) stability.

**Ceramics:** Requires 2.0 mol/L HNO₃ (pH ~1.0) to protonate Al₂O₃ sites and leach Mg²⁺ (Table 5).

**Comment 5: SEM Validation and Detection Methods**

**Reviewer’s Question:**

- Were AFM or other methods used to validate SEM observations? How was iodine-131 detection confirmed?

**Response:**

1. **SEM/AFM**: AFM was not performed due to equipment limitations, but SEM images show clear post-decontamination cleanliness (Figs. 4–5).

2. **Iodine-131 Validation:**

- EDS cannot detect trace iodine (concentration ~10⁻¹¹ mol/L).

- **Primary Method:** β-counting (LB-4 system, detection limit 0.05 Bq) cross-validated DF calculations (Section 2.1).

**Comment 6: Ce(IV) Stability and HNO₃ Role**

**Reviewer’s Question:**

Was Ce(IV) concentration monitored? Does HNO₃ directly participate in redox?

**Response:**

1. **Ce(IV) Monitoring:** Ce(IV)→Ce(III) reduction was inferred via kinetic studies (pseudo-first-order model, Table 11).

2. **HNO₃ Role:**

- Maintains Ce(IV) stability.

- Directly oxidizes iodine via:

*3I⁻ + NO₃⁻ + 4H⁺ → 3I₂ + NO↑ + 2H₂O* (Equation 2, Section 3.5).

**Comment 7: Kinetics and Material Composition**

**Reviewer’s Question:**

Could ceramics achieve similar DF at lower temperature/longer time? How does composition affect decontamination?

**Response:**

**1. Kinetics:**

- Ceramics require higher temperatures (80°C) for rapid iodine removal (kₒbₛ = 0.035 min⁻¹ vs. 0.021 min⁻¹ for quartz, Table 11).

- Lower temperatures prolong the process due to slower Al₂O₃ reactivity.

**2. Material Influence:**

- **Quartz**: Oxidative dissolution dominates.

- **Ceramics**: Acid-assisted ion exchange (Table 10).

**Comment 8: Organic Contaminants and Reproducibility**

**Reviewer’s Question:**

Were organics inherent or introduced? Were orthogonal tests repeated?

**Response:**

1. **Organic Contaminants**: Likely introduced during handling (SEM shows removal via Ce(IV)/•OH radicals, Section 3.5).

2. **Reproducibility**: Triplicate measurements showed RSD <5% (Table 6).

**Comment 9: DF Variability and Oxidant Choice**

**Reviewer’s Question:**

Are DF variations due to error or material response? Why Ce(IV) over permanganate/ozone?

**Response:**

1. **DF Variability**: Reflects true material-response differences (ANOVA F = 1.32, p > 0.05, Section 3.3).

2. **Ce(IV) Advantage:**

- Higher redox potential (+1.72 V) vs. permanganate (+1.51 V).

- Less corrosive than ozone (Refs. 23–25).

**Comment 10: pH and Kinetic Studies**

**Reviewer’s Question:**

Could pH adjustments optimize DF? Were kinetic models tested?

**Response:**

1. **pH Impact**:

- Optimal pH ranges: ~1.5 (quartz) and ~1.0 (ceramics) (Section 3.6).

- Outside these ranges, Ce(IV) hydrolyzes or iodine re-adsorbs.

2. **Kinetics**: Pseudo-first-order model confirmed temperature dependence (Table 11).

**Comment 11: SEM Quantification and Sample Size**

**Reviewer’s Question:**

Can SEM changes be quantified? How might larger samples affect DF?

**Response:**

1. SEM Quantification: Added qualitative descriptions of surface cleanliness (Figs. 4–5 captions).

2. **Scalability:** Pilot tests on larger samples (e.g., 300 mm ceramics) are planned (Section 4).

**Comment 12: Introduction Strengthening**

**Reviewer’s Question:**

Clarify the study’s novelty and contextualize with existing research.

**Response:**

Added three key innovations to the Introduction (Lines 24–36):

1. First quantitative framework for Ce(IV)/HNO₃ on quartz/ceramics.

2. Faster (1–2 h) and more efficient (DF up to 19.52) than conventional methods.

3. Mechanistic insights into material-specific HNO₃ dependence.

Cited suggested references (DOIs: 10.1021/acsapm.4c03794, 10.1007/s10967-024-09667-4, 10.3390/nano12132305).

**Conclusion**

We thank the reviewer for their constructive feedback. All changes have been incorporated into the revised manuscript, with tracked modifications and a clean version uploaded separately. We hope the revisions meet the journal’s standards.

Sincerely,

The Authors
